# Identifying the Question Similarity of Regulatory Documents in the Pharmaceutical Industry by Using the Recognizing Question Entailment System: Evaluation Study

**DOI:** 10.2196/43483

**Published:** 2023-09-26

**Authors:** Nidhi Saraswat, Chuqin Li, Min Jiang

**Affiliations:** 1 Eli Lilly and Company Indianapolis, IN United States

**Keywords:** regulatory affairs, frequently asked questions, FAQs, Recognizing Question Entailment system, RQE system, transformer-based models, textual data augmentations

## Abstract

**Background:**

The regulatory affairs (RA) division in a pharmaceutical establishment is the point of contact between regulatory authorities and pharmaceutical companies. They are delegated the crucial and strenuous task of extracting and summarizing relevant information in the most meticulous manner from various search systems. An artificial intelligence (AI)–based intelligent search system that can significantly bring down the manual efforts in the existing processes of the RA department while maintaining and improving the quality of final outcomes is desirable. We proposed a “frequently asked questions” component and its utility in an AI-based intelligent search system in this paper. The scenario is further complicated by the lack of publicly available relevant data sets in the RA domain to train the machine learning models that can facilitate cognitive search systems for regulatory authorities.

**Objective:**

In this study, we aimed to use AI-based intelligent computational models to automatically recognize semantically similar question pairs in the RA domain and evaluate the Recognizing Question Entailment–based system.

**Methods:**

We used transfer learning techniques and experimented with transformer-based models pretrained on corpora collected from different resources, such as Bidirectional Encoder Representations from Transformers (BERT), Clinical BERT, BioBERT, and BlueBERT. We used a manually labeled data set that contained 150 question pairs in the pharmaceutical regulatory domain to evaluate the performance of our model.

**Results:**

The Clinical BERT model performed better than other domain-specific BERT-based models in identifying question similarity from the RA domain. The BERT model had the best ability to learn domain-specific knowledge with transfer learning, which reached the best performance when fine-tuned with sufficient clinical domain question pairs. The top-performing model achieved an accuracy of 90.66% on the test set.

**Conclusions:**

This study demonstrates the possibility of using pretrained language models to recognize question similarity in the pharmaceutical regulatory domain. Transformer-based models that are pretrained on clinical notes perform better than models pretrained on biomedical text in recognizing the question’s semantic similarity in this domain. We also discuss the challenges of using data augmentation techniques to address the lack of relevant data in this domain. The results of our experiment indicated that increasing the number of training samples using back translation and entity replacement did not enhance the model’s performance. This lack of improvement may be attributed to the intricate and specialized nature of texts in the regulatory domain. Our work provides the foundation for further studies that apply state-of-the-art linguistic models to regulatory documents in the pharmaceutical industry.

## Introduction

### Regulatory Affairs

In a pharmaceutical company, the regulatory affairs (RA) department is responsible for obtaining approval for new pharmaceutical products and ensuring that approval is maintained for as long as the company wants to keep the product on the market. It serves as the interface between the regulatory authorities (such as the Food and Drug Administration, European Medicines Agency, etc) and pharmaceutical companies. It is the responsibility of the RA department to keep abreast of current legislation, guidelines, and other regulatory intelligence.

Regulatory data sources are dynamic and enormous. Regulatory professionals go through the extremely tedious and grueling task of extracting relevant information for various regulatory tasks. The process includes generating one or more suitable key phrases; searching for these key phrases in multiple data sources; and combining appropriate information retrieved from different data sources into a clear, compact, and concise summary of findings. Keeping track of such large data sources for relevant information manually is difficult and complex. An artificial intelligence–powered search system can drastically reduce manual efforts and improve the efficiency and quality of the existing processes.

The question answering (QA) system is an efficient approach for retrieving information. Much research has been conducted on open-domain QA systems based on deep learning techniques owing to the availability of vast data sources. However, the medical domain received less attention owing to the shortage of medical data sets. Although electronic health records empower the field of medical QA by providing medical information to answer user questions, the gap remains significant in the medical domain, especially for text-based sources.

The intricate challenges of automated QA in the biomedical domain are growing with the increasing diversity and specialization of medical texts. One of the promising tracks investigated in QA is to map new questions to formerly answered questions that are “similar.” Frequently asked questions (FAQ) component in an intelligent search system can considerably speed up the automated search system and enhance the status of search results. Therefore, an FAQ model component that interacts with the user query input to return a similar question that has already been asked in the recent past can significantly accelerate the remaining components of the search system pipeline and improve the system’s effectiveness.

In this study, we proposed a new approach for detecting similar questions based on Recognizing Question Entailment (RQE) in the RA domain. We considered FAQs as a valuable and widespread source of information.

RQE is a crucial component of modern QA systems. The RQE approach for a QA system is to retrieve answers to a given question proposed by users using natural languages by retrieving answers to an entailed and already answered question. The answered question and its associated answer are saved in a question-answer pair database. Question entailment is formally defined by Ben Abacha and Demner-Fushman [1] as follows: question A entails question B if every answer to question B is also a correct answer to question A exactly or partially. It is a challenging task to understand questions and judge the semantic similarity of two questions: (1) one question could be rephrased in many different ways and (2) two different questions may refer to the same problem and could be answered by the same answer [2].

### Background

#### RQE in the General Domain

Researchers in the general domain used 2 public benchmark data sets for question similarity tasks: SemEval and Quora question pair. These 2 data sets have labeled training data for question-question similarity. SemEval-2017 task 3 [3] featured questions from subforums of StackExchange, a family of technical community support forums. Quora question pair data set contains pairs of similar questions asked by people on the Quora website. The topics of these questions range from philosophy to entertainment. The best-performing systems for the SemEval question similarity task used syntactic tree kernels or the SoftCosine metric [4]. Kunneman et al [5] compared 2 recent approaches (SoftCosine and Smoothed partial tree kernel) and 2 traditional approaches (BM25 [6] and translation-based language model) and showed that the choice of a preprocessing method and a word-similarity metric have a considerable impact on the final result. Shah et al [7] first applied the adversarial domain adaptation to the problem of duplicate question detection across different domains and outperformed the best baseline on StackExchange questions. More recently, Nguyen et al [8] outperformed previous studies on the SemEval data set by combining a convolutional neural network and features from external knowledge to measure the similarity between 2 questions. In addition to these studies on the aforementioned popular data sets, Wang et al [9] used a method based on the Coattention-DenseGRU (gated recurrent unit) to match similar questions on Chinese rice-related questions.

Although many researchers have put efforts into recognizing general question similarity, their approaches do not generalize well to domains that require domain expert knowledge, such as the biomedical domain. First, questions in the biomedical domain demand much domain-specific knowledge, and a single word can change the meaning of the question [10]. Second, there are few publicly available biomedical question–question similarity data sets, resulting in a limited number of samples that can be used to train models that can effectively learn those differences. Given the increasing popularity of RQE-based QA systems, question similarity in the biomedical domain is currently an active research area. A growing number of RQE-based QA systems have been proposed, and an international challenge was held in 2019 [11].

#### RQE in Biomedical Domain

A wide range of approaches has been proposed to capture the semantic relationship between pairs of questions in RQE-based QA systems. Luo et al [12] calculated similarities between questions using statistical syntactic features and Unified Medical Language System annotated semantic features. Ben Abacha and Demner-Fushman [1] used machine learning models with lexical features and semantic features to determine the similarity of question pairs. More recent studies have gone beyond traditional feature-based methods and used deep learning models. Wang and Nyberg [13] used a dual entailment approach with bidirectional recurrent neural networks and attention mechanisms to predict question similarity. Ben Abacha and Demner-Fushman [14] improved their system using feature-based logistic regression and neural network that passed the concatenated sentence representations to multiple ReLU layers to classify question pairs into entailment or no entailment categories. McCreery et al [10] augmented a general language model with medical knowledge by using a double fine-tuning process. A pretrained language model is first fine-tuned with a large general corpus (eg, Quora question pairs) and then fine-tuned with a small number of labeled question pairs.

The MEDIQA 2019 challenge [11] included 3 tasks: natural language interface (NLI), RQE, and QA in the medical domain. It aimed to further research efforts to improve domain-specific information retrieval and question-answer systems. In the challenge, approaches using ensemble methods and transfer learning of multitask language models outperformed traditional deep learning models for RQE task [11]. The PANLP team [15] achieved the best result on RQE task by fine-tuning the pretrained language models, Bidirectional Encoder Representations from Transformers (BERT) [16] and multitask–deep neural network (DNN) [17]. They further boosted the performance on the RQE task by transfer learning from the NLI task. The Sieg team [18] ranked second for RQE tasks and used a multitask learning approach, with shared layers trained for the NLI on the RQE task. Approaches that used ensemble methods without multitask language models [19] ranked third in the competition, and approaches that used multitask models without ensemble methods [20] ranked fourth. More recently, Sarrouti et al [21] proposed a multitask transfer learning method based on data augmentation for RQE. They outperformed other teams on the RQE test set of the 2019 MEDIQA challenges.

RQE or similarity is part of another more general natural language processing (NLP) task called semantic textual similarity (STS). Tasks of STS include comparing 2 sentences, 2 paragraphs, or even 2 documents. RQE is more closely related to QA and information retrieval systems.

#### STS in the General Domain

STS is connected to textual entailment (TE) and paraphrasing; however, it differs in many ways and is more directly applicable to several NLP tasks. Semantic similarity or STS is a task in NLP that scores the relationship between texts or documents using a defined metric. The aim is to identify the likeness or similarity in the meaning of 2 pieces of text.

STS differs from TE in that it assumes bidirectional graded equivalence between a pair of textual snippets. In the case of TE, the equivalence is directional; for example, a car is a vehicle, but a vehicle is not necessarily a car. STS also differs from both TE and paraphrasing in that rather than being a binary yes-or-no decision (eg, a vehicle is not a car), we defined STS to be a graded similarity notion (eg, a vehicle and a car are more similar than a wave and a car).

#### STS in the Biomedical Domain

STS in the clinical domain can empower stakeholders to detect and eliminate redundant information that may reduce the cognitive burden and improve the clinical decision-making process. The description in the study by Wang et al [22] discusses the details of the task of identifying clinical STS (ClinicalSTS). The participating systems were asked to return a numerical score, ranging from 0 to 5, indicating the degree of semantic similarity between the pair of 2 clinical sentences. The performance was measured using the Pearson correlation coefficient between the predicted similarity scores and human judgments.

1. The winning team submitted 4 systems. The first system was the random forest model using 63 features including string similarity features, entity similarity features, number similarity features, and deep learning features. The second system used the average score of the first system and dense neural networks. The third system, which was also the best-performing system among all submitted systems with a Pearson correlation of 0.8328, applied a regression model on 8 trained models including the random forest model, the Bayesian Ridge regression model, the Lasso regression model, the linear regression model, the Extra Tree model, the DNN using the Universal Sentence Encoder, the DNN using the inferSent encoder, and the Encoder–multilayer perceptron using the inferSent encoder. The fourth system used the average score of the first system, and the Encoder–multilayer perceptron used the inferSent encoder.
2. The team that placed second in this challenge used attention-based convolutional neural network (ABCNN) and bidirectional long short-term memory (Bi-LSTM) networks. One of their submissions used ABCNN with traditional NLP features. The second is a hybrid model of ABCNN and Bi-LSTM, with traditional NLP features. The third run ensembled the previous 2 systems by calculating the average scores. The ensemble model performed the best among their submitted systems.
3. The third-placed team proposed a sentence-embedding method that represents a sentence as a weighted average of word vectors, followed by a soft projection. They used a self-regularized identity map named Conceptors to correct the common component bias in linear sentence embedding. Majority voting and 2 different support vector regression models with only word embedding representation features were explored by the fourth-placed team for their submissions. The best performance was achieved by the majority voting method.

Lastra-Díaz and García-Serrano [23] presented an empirical study on the impact of a number of model design choices on a BERT-based approach to clinical STS. It was demonstrated that the proposed hierarchical convolution mechanism outperformed several alternative conventional pooling methods. Different parameter fine-tuning strategies with varying degrees of flexibility were investigated, and the optimal number of trainable transformer blocks was identified, thereby preventing overtuning. Finally, the utility of 2 data augmentation methods (segment reordering and back translation) on clinical STS was verified.

Hadj Taieb et al [24] proposed a novel framework based on a gated network to fuse distributed representation and one-hot representation of sentence pairs. Some state-of-the-art distributed representation methods, including convolutional neural network, Bi-LSTM, and BERT, were used in this framework, and a system based on this framework was developed for a shared task regarding clinical STS organized by BioCreative and OHNLP in 2018.

Elavarasi et al [25] demonstrated transformer-based models (BERT, XLNet, and RoBERTa) and developed a system that can use various transformer algorithms for measuring clinical STS. STS system has two modules: (1) a transformer model–based feature learning module that learns distributed sentence-level representations from sentence pairs and (2) a regression-based similarity score learning module that calculates similarity score between 0 and 5 according to the distributed representations derived from the transformers. The authors explored several methods to combine the distributed representations from different transformers, including (1) simple head-to-tail concatenation, (2) pooling, and (3) convolution. The experiment’s results showed that the RoBERTa model achieved the best performance compared with other transformer models.

The work done in the study by Lastra-Díaz et al [26] focuses on ranking the degree of similarity between clinical texts. The paper studied the impact of using different preprocessing methods as well as different feature representation methods (word embeddings–BioWordVec vs sentence embeddings–BioSentVec) by proposing a system with a simple neural network. The study demonstrated that sentence embeddings provided superior text representation than word embeddings, better capturing sentence semantics, whereas word embeddings were not a distant performer. It was observed that word embeddings benefited from using a more thorough text-preprocessing pipeline, whereas sentence embeddings obtained better test results with a basic preprocessing approach.

#### Data Sets for STS

This subsection briefly describes some of the popular data sets at the sentence pairs level that are used to evaluate the semantic similarity algorithms. The performance of various semantic similarity algorithms is measured by the correlation of the achieved results with that of the standard measures available in these data sets. Li et al [27] used a data set comprises 65 sentence pairs that were created using the dictionary definition of 65 word pairs used in the Rubenstein-Goodenough data set [28]. A similarity range of 0 to 4 (from the lowest to the highest) was provided voluntarily by 32 native English speakers. The mean of the scores given by all the volunteers was taken as the final score. The SICK data set [29] consists of 10,000 sentence pairs derived from 2 existing data sets, the ImageFlickr 8 and MSR-Video descriptions data sets. Each sentence pair is associated with a relatedness score and a text entailment relation. The relatedness score ranges from 1 to 5, and the 3 entailment relations are “NEUTRAL, ENTAILMENT, and CONTRADICTION.” The annotation was performed using crowdsourcing techniques. The STS [30-34] data sets were built by combining sentence pairs from different sources by the organizers of the SemEVAL shared task. The data set was annotated using Amazon Mechanical Turk and verified by the organizers themselves. Various sources such as newswire, videos, glosses, Workshop on Machine Translation evaluation, Machine Translation evaluation, newswire headlines, forum posts, news summary, image descriptions, tweet news pairs, student answers, QA forum answers, and committed belief were used to build the STS data set.

The computation of semantic similarity between various types of text fragments such as words, sentences, or documents plays a key role in a wide range of NLP tasks such as information retrieval [35], text summarization [36], text classification [37], essay evaluation [38], machine translation [39], and QA [40,41].

A wide range of semantic similarity measures has been proposed and applied in various applications and domains. These measures vary in performance based on their approaches and application domains. Detailed comparisons of these measures can be found in previous work [22,42-47].

Amir et al [42] proposed a semantic similarity algorithm using kernel functions. They used constituency-based tree kernels where the sentence is broken down into subject, verb, and object based on the assumption that most semantic properties of a sentence are attributed to these components. The input sentences are parsed using the Stanford Parser to extract various combinations of subject, verb, and object. The similarity between the various components of the given sentences is calculated using a knowledge base, and different averaging techniques are used to average the similarity values to estimate the overall similarity, and the best among them is chosen based on the root mean squared error value for a particular data set. Benedetti et al [43] proposed a novel knowledge-based technique, Context Semantic Analysis, for estimating interdocument similarity. The technique is based on a Semantic Context Vector, which can be extracted from a knowledge base and stored as metadata of a document and used to compute interdocument similarity. The authors also demonstrated how Context Semantic Analysis can be effectively applied in the information retrieval domain, even if user queries, typically composed of a few words, contain a limited number of entities. Yang et al [44] presented a response prediction model that learns a sentence encoder from conversations. The encoder learned from the input-response pairs performs well on sentence-level STS. The basic conversation model learned from Reddit conversations is competitive with existing sentence-level encoders on public STS tasks. A multitask model trained on Reddit and Stanford NLI classification achieved the state-of-the-art for sentence encoding–based models on the STS Benchmark task. An FAQ retrieval system with a method using query-question similarity and BERT-based query answer relevance was proposed by Sakata et al [48]. A traditional unsupervised information retrieval system is used to calculate the similarity between the query and the question. In contrast, the relevance between the query and answer, calculated using BERT model, are learned using QA pairs in an FAQ database. Minaee and Liu [49] evaluated the proposed approach on two data sets: (1) localgovFAQ, a data set that is constructed in a Japanese administrative municipality domain, and (2) StackExchange data set, which is the public data set in English. Uva et al [50] proposed to inject structural relationships in neural networks by (1) learning a support vector machine model using tree kernels on relatively few pairs of questions (a few thousands), as gold standard training data are typically scarce; (2) predicting labels on a very large corpus of question pairs; and (3) pretraining neural networks on such a large corpus. The experiments in the study were performed on the Quora and SemEval question similarity data sets. A deep learning–based model for automatic QA was proposed [51] to solve the use case of customer case service automation. The questions and answers are embedded using neural probabilistic modeling (doc2vec), followed by training a deep similarity neural network to determine the similarity score of a pair of answer and question. For each question, the best answer is found as the one with the highest similarity score. Cai et al [51] trained this model on a large-scale public QA database and then fine-tuned it to transfer to the customer care chat data.

#### About Transfer Learning

The advancements of deep learning in NLP in recent years have improved, accelerated, and automated various functions and features of text analytics. Deep learning enables models to understand and learn the meaning of words and phrases in different language contexts. However, all these utilities demand large and complex deep learning models that are data hungry. They require training with thousands or millions of data points before making a plausible prediction. Training is expensive in terms of both time and resources. The issue with such models is that they are performed only on a single task. Future tasks require a new set of data points and a greater number of resources. Transfer learning comes into the picture by transferring knowledge learned from one model to another.

Transfer learning is a machine learning method where a model trained on one task is repurposed on a second related task as an optimization that allows rapid progress when modeling the second task. It can train DNNs with comparatively fewer data.

We subsequently briefly describe a few DNN models experimented with in this paper that use the transfer learning approach.

#### BERT

Google’s BERT [16] has significantly altered the NLP landscape in recent years. BERT is a contextualized word representation model based on a masked language model and pretrained using bidirectional transformers. It is designed to pretrain deep bidirectional representations from the unlabeled text by jointly conditioning on both the left and right context. As a result, the pretrained BERT model can be fine-tuned with only one additional output layer to create state-of-the-art models for a wide range of NLP tasks.

BERT is pretrained on a large corpus of unlabeled text, including the entire Wikipedia (2500 million words) and Book Corpus (800 million words). BERT is a “deeply bidirectional” model, meaning that BERT learns information from both the left and the right side of a token’s context during the training phase.

BERT architecture builds on top of the transformer. all these transformer layers are encoder-only blocks. BERT is pretrained on 2 NLP tasks: masked language modeling and next-sentence prediction. The pretrained BERT has a maximum of 512 input tokens (position embeddings). The output would be a vector for each input token. Each vector is composed of 768 float numbers (hidden units).

#### Clinical BERT

BERT model is pretrained in general text corpora. A specific model pretrained on specialty corpora, such as clinical text, is available in the form of Clinical BERT, a modified BERT model. Specifically, the representations are learned using medical notes and further processed for downstream clinical tasks. Clinical BERT [52] models are pretrained on 2 types of data: one for generic clinical text and another for discharge summaries. Similar to BERT, Clinical BERT is a trained transformer encoder stack. Clinical BERT is also a bidirectional transformer.

#### BioBERT

BioBERT [53] is a domain-specific language representation model that is pretrained on large-scale biomedical corpora. BioBERT is specifically pretrained on PubMed abstracts (PubMed) and PubMed Central full-text articles along with English Wikipedia and Book Corpus data sets as in BERT.

#### BlueBERT

The success of the General Language Understanding Evaluation, which was primarily to help the development of pretrained language models based on performance on generic NLP tasks, led to the development of Biomedical Language Understanding Evaluation (BLUE). BLUE is similar to General Language Understanding Evaluation but is more specific to the biomedical domain. The benchmark consists of 5 tasks with 10 data sets covering biomedical and clinical texts with different data set sizes and difficulties. BlueBERT [54], which was originally named National Center for Biotechnology Information BERT, was pretrained on PubMed abstracts and MIMIC-III (Medical Information Mart for Intensive Care) clinical notes. The work done by Peng et al [54] focused on experimenting BLUE in conjunction with Embeddings from Language Model and BERT models. BlueBERT was found to be the best-performing model and significantly superior to other models in the clinical domain.

Table 1 summarizes the pretraining details of different BERT models used in the experiments of this study.

**Table 1:** Summary of pretraining details for the various Bidirectional Encoder Representations from Transformers (BERT) models used in our experiments.

Model	Vocabulary	Pretraining	Corpus	Text size
BERT	Wikipedia+Books	N/A^a^	Wikipedia+Books	3.3B words (16 GB)
Clinical BERT	Wikipedia+Books	Continual pretraining	MIMIC^b^ (subset)+MIMIC-III	0.5B words (3.7 GB)
BioBERT	Wikipedia+Books	Continual pretraining	PubMed+PMC^c^	4.5B words
BlueBERT	Wikipedia+Books	Continual pretraining	PubMed+MIMIC-III	4.5B words

^a^N/A: not applicable.

^b^MIMIC: Medical Information Mart for Intensive Care.

^c^PMC: PubMed Central.

## Methods

### DNN Architecture

**Figure 1:**
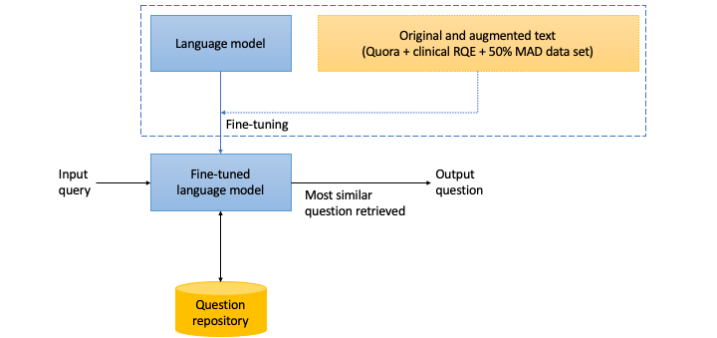
Architecture diagram for frequently asked questions (FAQ) system. MAD: manually annotated data set; RQE: Recognizing Question Entailment.

Figure 1 describes the working of our proposed FAQ system. The proposed FAQ system uses 2 major components: a question repository and a fine-tuned language model. The FAQ repository, which acted as the source of questions to identify entailment or no entailment for input queries, was maintained in the proposed FAQ system. The input query to the fine-tuned language model was compared against each question in the question repository to identify and retrieve the most similar FAQ, if any.

The language model was fine-tuned by using the Quora question pairs and clinical RQE (C-RQE) data sets. Different experimental and data split strategies were used to identify the best-performing model configuration. These data sets and experimental strategies are explained in detail in the following subsections.

### Data Sets

The experiments in this paper were based on these three different data sets.

1. *Quora questions pairs (Quora)*: The Quora question pairs data set [55] provides an opportunity to train and test models of semantic equivalence, based on actual Quora data. Each line in the data set contains an ID for each question in the question pair, a unique ID for the question pair, the full text for each question, and a binary label that indicates whether the line contains a duplicate question pair. Table 2 presents a few sample lines of the data set. This data set contains over 400,000 labeled question pairs. Of the 404,290 question pairs, 255,027 (63.08%) had a negative (0) label and 149,263 (36.92%) had a positive (1) label, making our data set unbalanced.
2. *C-RQE*: The work done in the study by Ben Abacha and Demner-Fushman [1] describes an automatic method for constructing training corpora for RQE. The RQE data set constructed in this paper used the National Library of Medicine collection of 4655 clinical questions asked by family physicians. The resulting C-RQE data set had approximately 8588 question pairs in the form of an XML, with RQE value labels as true or false.
3. *Regulatory RQE—manually annotated data set (MAD):* The subject matter experts, who are part of the organizational RA team, manually annotated a collection of 268 question pairs with entailment and no entailment labels. Of these 268 question pairs, 127 were entailment pairs and 141 were no entailment pairs. The records in this data set were of the following format: (question_pair_ID, label, question1, question2). Some of the example records from this data set are presented in Table 3.

**Table 2:** Samples of Quora question pairs.

ID	Question1 ID	Question2 ID	Question1	Question2
447	895	896	What are natural numbers	What is a least natural number?
1518	3037	3038	Which pizzas are the most popularly ordered pizzas on Domino's menu?	How many calories does a Domino’s pizza have?
3272	6542	6543	How do you start a bakery?	How can one start a bakery business?
3362	6722	6723	Should I learn python or Java first?	If I had to choose between learning Java and Python, what should I choose to learn first?

**Table 3:** Samples of question pairs in the test set.

ID	Label	Question 1	Question 2
1	Entailment	Is Emend approved by the European Medicines Agency?	Has the European Medicines Agency authorized Emend?
2	Entailment	What is Emend approved indication in the European Union?	What is the indication of Emend in the European Union product information?
15	Not_entailment	Does Tisagenlecleucel has gotten an orphan designation by the European Medicines Agency?	Does Tisagenlecleucel refused by the European Medicines Agency?
16	Not_entailment	Is ELIANA (other IDs: NCT02435849/CCTL019B2202) a single arm trial?	Is ELIANA (other IDs: NCT02435849/CCTL019B2202) a randomized arm trial?

### Preprocessing of Data Sets

Both the Quora and C-RQE data sets were transformed to a format that was consistent with the MAD data set. The Quora data set was transformed to this format by removing individual question IDs and converting “is_duplicate” binary field to “entailment/no entailment” label field (“is_duplicate=1” indicates entailment label and vice versa). In contrast, the C-RQE data set, which is an XML, was converted to the format consistent with MAD by extracting ID, question1, question2, and value labels. The “value label=true” was transformed into an entailment label and vice versa.

### Data Split Strategy

Three data sets are commonly used in deep learning model development: training, validation, and test sets. The model is trained on the training set, and the validation set is used to evaluate the model fit unbiasedly during the hyperparameter tuning stage. The test set is independent from the training and validation sets and is used to assess the model’s performance*.*

The experiments designed in this study are built on 2 types of data set split strategy described as follows:

*Strategy 1:* Quora and C-RQE data sets were used as training and validation sets, respectively. With this strategy, we have 404,283 sentence pairs in the training data set, 7143 pairs in the validation data set, and 150 pairs in the testing data set.

*Strategy 2:* Quora and C-RQE data sets were combined to further split them into training and validation sets such that the training set had approximately 90% of the records, whereas the remaining 10% were part of the validation set. Therefore, the training set had 90% of the records from Quora and C-RQE data sets. The validation set comprised 10% of the records from Quora and C-RQE data sets, which were not part of the training set. The validation set also included 50% of MAD, which was not part of the test set. With this strategy, we have 370,282 sentence pairs in the training data set, 41,279 pairs in the validation data set, and 150 pairs in the testing data set.

### Model Evaluation

The variation of experiments conducted in this study for the Quora, C-RQE, and MAD data sets were performed on top of 4 types of BERT models: (1) regular BERT [16], (2) Clinical BERT [52], (3) BioBERT [53], and (4) BlueBERT [54]. The performance of several types of model configurations was evaluated for accuracy, *F*_1_-score metrics, and the area under the receiver operating characteristic curve (AUC). The model’s accuracy was estimated by finding the total number of true entailment or no entailment predictions out of the total number of predictions done by the model. *F*_1_-score was an error metric that was calculated from the precision and recall of the test. *F*_1_-score of the model was interpreted as the harmonic mean of the precision and recall, conveying the balance between the precision and recall of the model.



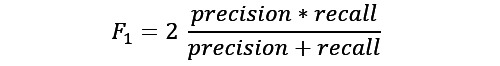



*F*_1_-score ranges from 0 to 1, with 0 being the worst and 1 being the best value. The highest value of 1 indicates that the model has a perfect precision and recall, whereas the lowest value of 0 indicates that either the precision or recall is 0.

AUC is another commonly used statistical metric that evaluates the performance of classification models and provides a comprehensive measure of a model’s ability to classify instances from different classes correctly. The AUC metric has advantages over accuracy and *F*_1_-score in that it is insensitive to data imbalance and considers the model’s behavior across all possible classification thresholds. The AUC value lies in the range of 0 to 1, where a higher value indicates a more robust ability for classification. An AUC of ≤0.5 suggests a model with no predictive power other than random guessing.

### Experimental Design

The experiments performed in this paper used regular BERT, Clinical BERT, BioBERT, and BlueBERT models, which were fine-tuned on the Quora, C-RQE, and MAD data sets. Each of these models was fine-tuned on the 2 data split strategies. For experimentation of regular BERT, we used bert-base-uncased, whereas the Clinical BERT model used in this paper was pretrained on clinical notes. The BlueBERT-Base, Uncased, PubMed+MIMIC-III variant of the BlueBERT model was experimented with in this paper.

We used the Hugging face transformers library for model fine-tuning and text classification. The following set of hyperparameters was established to be the best set of hyperparameters and was used for all the experiments conducted in this paper: epochs=3, learning rate=3 × 10^–5^, batch size=32, and maximum sequence length=150.

### Ethical Considerations

This study is not human participant research; thus, no ethics approval was sought.

## Results

The experimental design of this paper is described in Table 4.

Table 4 describes the performance of different BERT models on the datasets discussed in the *Data Split Strategy* section. As baseline experiments, we used experiments 1, 4, 7, and 10 to assess the performance of the models without transferring any prior knowledge. With comparable accuracy, *F*_1_-score, and AUC, the Clinical BERT and BioBERT models outperformed the other 2 baseline models, whereas the BlueBERT model performed the lowest with an accuracy of 48.9%, *F*_1_-score of 0.328, and AUC of 0.242.

**Table 4:** Performance of different Bidirectional Encoder Representations from Transformers (BERT) models on data sets: without augmentation.

Experiment	Model	Data split strategy	Accuracy (%)	*F*_1_-score	AUC^a^
1	BERT	N/A^b^	48.9	0.328	0.303
2	BERT	1	82	0.808	0.920
3	BERT	2	90.66	0.904	0.958
4	Clinical BERT	N/A	58.6	0.586	0.584
5	Clinical BERT	1	90	0.894	0.971
6	Clinical BERT	2	90	0.897	0.961
7	BioBERT	N/A	54.1	0.513	0.612
8	BioBERT	1	66	0.538	0.729
9	BioBERT	2	56.66	0.515	0.580
10	BlueBERT	N/A	48.9	0.328	0.242
11	BlueBERT	1	82	0.807	0.920
12	BlueBERT	2	84.66	0.842	0.920

^a^AUC: area under the receiver operating characteristic curve.

^b^N/A: not applicable.

Regardless of the data split approach, the performance of all models enhanced after being fine-tuned with domain-specific data. BERT’s accuracy, *F*_1_-score, and AUC improved the most after being fine-tuned with data split strategy 1. The accuracy of the model increased from 48.9% to 90.66%. The performance of BioBERT model showed minimal improvement. The accuracy of the BioBERT model increased from 54.1% to 66% after being fine-tuned with our data split strategy 1. Although the accuracy of BioBERT model improved from 54.1% to 56.66%, the model’s classification capability decreased because AUC decreased from 0.612 to 0.580.

The best-performing models were BERT (data split strategy 2) and Clinical BERT (data split strategy 1 and 2) with an accuracy of 90.66%, 90%, and 90%; *F*_1_-score values of 0.904, 0.894, and 0.897; and AUC of 0.958, 0.971, and 0.961, respectively. Experiments 1 and 2 used the general BERT model to provide 82% and 90.66% accuracy for data split strategy 1 and 2, respectively. This behavior of data split strategy 2 surpassing data split strategy 1 was consistent across all BERT models experimented in Table 4, except for the BioBERT model. The Clinical BERT model with both data split strategies was among the top-performing models with an accuracy of approximately 90% and an AUC of >0.96. The BioBERT model did not fare very well compared with all the other models in Table 4, with an accuracy of 66% and 56.66% for data split strategy 1 and 2, respectively. The BlueBERT model performed noticeably better than BioBERT, with an accuracy of approximately 82% and 84.66% for data split strategy 1 and 2, respectively, which was still lower than that of the high-performing BERT and Clinical BERT models.

## Discussion

### Principal Findings

In this study, we used computational models to recognize question entailment in pharmaceutical regulatory domains. As there is no publicly available labeled data set in this field, we adopted the idea of transfer learning. We fine-tuned 4 different versions of pretrained BERT language models on 2 publicly available data sets (Quora and C-RQE). The best model achieved 90.66% accuracy in RQE on our MAD, which contained 150 question pairs in the regulatory field. To the best of our knowledge, this study is the first to use state-of-the-art NLP models to recognize question semantic similarity in the pharmaceutical regulatory domain. Our study could provide the foundation for future studies that apply NLP technologies to text in the pharmaceutical regulatory domain.

As shown in Table 4, the BERT model outperformed the other BERT variants in terms of its ability to learn domain knowledge using transfer learning. Although the BERT model performed poorly on the test data set before fine-tuning, its accuracy increased in RQE after being fine-tuned using domain-specific question pairs. This finding was also supported by experiments 2 and 3. The model based on BERT did not perform well with our data split strategy 1 (experiment 2). However, it reached the highest accuracy when we fine-tuned the BERT with our data split strategy 2 (experiment 3). Our data split strategy 1 used only Quora’s general domain question pairs as training resources. In contrast, strategy 2 includes both general domain question pairs and clinical questions from C-RQE as part of the training and validation sets. This indicates that BERT can perform well in the pharmaceutical regulatory domain text if we provide sufficient clinical domain background knowledge to the model and fine-tune it.

We also found that Clinical BERT models outperformed other BERT variants in this specific domain. Clinical narratives from general and nonclinical biomedical text have known differences in linguistic characteristics [52]. All BERT variants used in this study were initialized from BERT, but they were pretrained on the corpus from different fields. The Clinical BERT model was pretrained with clinical notes, the BioBERT model was pretrained with biomedical corpus, and the BlueBERT model was pretrained with the combination of biomedical text and clinical notes. We found that the Clinical BERT and BlueBERT models performed better than the BioBERT model. In other words, the models that were pretrained with clinical notes from MIMIC-III data set have a better performance than the models pretrained with PubMed articles in our RQE task. A possible reason is that the nature of questions in the regulatory domain, shown in Table 3, resonates more closely with the clinical notes text genre. This finding highlights the importance of pretraining with the proper text genre in learning the context-dependent representation [54].

Although DNNs perform well in a variety of NLP tasks, a large number of data are required to train deep learning models. The lack of training data has become one of the significant challenges to training deep learning models in the biomedical field, which could lead to underfitting models and could reduce their performance. We do not have a publicly available labeled data set for the pharmaceutical regulatory domain. Instead, we fine-tuned pretrained language models on the C-RQE data set to learn domain-specific knowledge. In our previous experiments, only 21% of the question pairs in the training corpus were from the regulatory-related domain. Consequently, we extended our experiments by expanding our training data set with data augmentation technologies. We aimed to study the impact and utility of augmentation techniques on pharmaceutical domain text using the general BERT and Clinical BERT models.

Researchers in the field of computer vision commonly use data augmentation to expand the number and variety of data without collecting new data. They create new image samples by rotating, changing the color, cropping, and compressing the images. Unlike images, the data in NLP are discrete, making it more challenging to generate high-quality augmented examples efficiently and effectively in the field of NLP.

With the increasing interest in and demand for data augmentation in NLP, many text data augmentation technologies have been proposed. Back translation is the most popular data augmentation method. The back translation approach involves translating a sequence into another language and then back to the original language. Deep learning models, such as Seq2Seq [56], neural machine translation [57], and transformers [58], can be used to translate. Various rule-based techniques have also been used in data augmentation. Wei and Zou [59] proposed Easy Data Augmentation, including synonym replacement, random insertion, deletion, and swapping. For paraphrase identification, Chen et al [60] built a signed graph over the data, with each sentence as nodes and labels as edges. They used balance theory and transitivity to induce augmented sentence pairs based on the graph. Kang et al [61] extended the Easy Data Augmentation method for biomedical named entity recognition by incorporating the Unified Medical Language System knowledge. Another class of techniques uses multiple samples to generate new pieces, pioneered by MixUp [62], which interpolates the inputs and labels of ≥2 examples. The difficult part of using MixUp in NLP is that it requires a continuous input. This issue was overcome by Chen et al [63], who mixed embeddings or higher layers. Some other model-based approaches used the text generation models, such as GPT-2 [64], to generate candidate examples from the training data set. Some trade-offs should be considered when choosing from these methods.

We used 2 data augmentation techniques in this study, entity replacement and back translation. The entity replacement technique in this study used the Scispacy [65] named entity recognition model trained on the BC5CDR corpus to identify CHEMICAL and DISEASE entities from the question pairs. The identified CHEMICAL and DISEASE entities were further replaced by synonyms from the dictionary of concepts and synonyms created from Observational Medical Outcomes Partnership (OMOP) Common Data Model. OMOP has consolidated multiple vocabularies into a common format, and OMOP’s Standardized Vocabularies contain all the code sets, terminologies, vocabularies, nomenclatures, lexicons, thesauri, ontologies, taxonomies, classifications, abstractions, and other such data that are required. This saves researchers and developers from having to understand and handle multiple formats and conventions of the originating vocabularies. For back translation, we used Google Translate application programming interface to do back translation and Chinese as the middle language. We compared several middle languages and found that Chinese had the best performance in recognizing question similarity. The original source text and back-translated text were compared to find differences, if any, in which case the back-translated text was used as an augmented record. In these experiments, we used only BERT and Clinical BERT as our base language models because these 2 models were found to have the best performance on the original test data set.

The results of the experiments with the augmented training data are shown in Table 5. We found that the data augmentation techniques did not improve the model’s performance. Experiments with back translation–augmented data samples performed better than experiments with entity replacement–augmented data samples. By analyzing the augmented data samples, we found that although these 2 data augmentation techniques expanded the number of data samples, they introduced some noise samples to our training set. This could be explained by the complexity and specificity of the text in the regulatory domain.

**Table 5:** Model performance with augmented training data.

Model	Data split strategy	Accuracy (%)		
		Entity replacement	Back translation	Entity replacement+back translation
BERT^a^	1	79.33	77.33	79.33
BERT	2	79.33	85.33	77.33
Clinical BERT	1	88.66	86.66	82.66
Clinical BERT	2	84	89.33	88

^a^BERT: Bidirectional Encoder Representations from Transformers.

Our study has some limitations. First, we only experimented with the BERT-based model in this study. Some other state-of-the-art pretrained language models, such as XLNet, T5, and GPT-2, also perform well in related NLP tasks. We will try other state-of-the-art models in our future studies. Second, we only had 150 pairs of questions in our test data set. If we had had a greater number of question pairs in our test data set, we would have better understood the performance of each model. Third, our manually labeled data set covers only a limited number and types of concepts in the regulatory domain. We should further our analysis by expanding the variety of question pairs.

### Conclusions

This study used deep learning models to recognize question entailment in the pharmaceutical regulatory domain. As no previous study has used computational models to learn text in the regulatory domain, our study demonstrates the possibility of using state-of-the-art artificial intelligence–based NLP models to understand the regulatory text. We also attempted 2 data augmentation techniques, back translation and entity replacement, to increase the number of training samples. However, these 2 techniques did not improve the model’s performance in this study.

## Abbreviations

ABCNN: attention-based convolutional neural network
AI: artificial intelligence
AUC: area under the receiver operating characteristic curve
BERT: Bidirectional Encoder Representations from Transformers
Bi-LSTM: bidirectional long short-term memory
BLUE: Biomedical Language Understanding Evaluation
C-RQE: clinical Recognizing Question Entailment
DNN: deep neural network
FAQ: frequently asked questions
MAD: manually annotated data set
MIMIC-III: Medical Information Mart for Intensive Care
NLI: natural language interface
NLP: natural language processing
OMOP: Observational Medical Outcomes Partnership
QA: question answering
RA: regulatory affairs
RQE: Recognizing Question Entailment
STS: semantic textual similarity
TE: textual entailment

## Data Availability

The Quora questions pairs data set [55] and clinical Recognizing Question Entailment data set [1] are publicly available. The regulatory Recognizing Question Entailment manually annotated data set generated during and analyzed during this study are not publicly available because of our company’s data protection and confidentiality policy but are available from the corresponding author on reasonable request.
